# Green Radiative Cooling and Antimicrobial Materials Based on Polylactic Acid Oligomer Hexagonal Microspheres

**DOI:** 10.3390/polym18212735

**Published:** 2026-11-03

**Authors:** Ziheng Zhang, Jun Li, Linlin Ma, Shirui Liu, Shunxiang Xu, Jun Hu

**Affiliations:** 1 College of Chemical Engineering, Zhejiang University of Technology, Hangzhou 310014, China; 2 ZJUT Yinhu Research Institute of Innovation and Entrepreneurship, Hangzhou 311400, China; 3 School of Environmental and Chemical Engineering, Wuyi University, Jiangmen 529020, China; 4 School of Fashion and Textiles, The Hong Kong Polytechnic University, Hong Kong 999077, China; 5 Department of Orthopaedics & Traumatology, Li Ka Shing Faculty of Medicine, The University of Hong Kong, Hong Kong 999077, China; shaunxu@hku.hk

**Keywords:** radiative cooling, antimicrobial materials, polylactic acid oligomer, hierarchically porous materials, microstructure

## Abstract

Thermal management relying on passive radiative cooling materials can result in remarkable energy savings and greenhouse gas emissions reductions compared with traditional air conditioner-based cooling. However, few of these materials are degradable; thus, their disposal may generate a new environmental problem. At the same time, the COVID-19 pandemic and other infectious diseases have raised requirements for hygienic and antimicrobial performance. In this work, we use a facile solvent evaporation method to prepare a novel hierarchically porous polylactic acid oligomer (PLAO) coating that exhibits a radiative cooling effect, broad-spectrum antimicrobial properties, and degradability (with a degradation rate of 99.5% after 28 days). The radiative cooling effect is attributed to the hexagonal microsphere geometry and nanotexture of the polylactic acid oligomer (PLAO) crystals, which enhance reflectivity in the solar spectrum through a multiple scattering effect and exhibit high emissivity in the mid-infrared atmospheric window. The hierarchically porous PLAO coating material achieves an apparent temperature reduction of 6.8 °C under solar-spectrum irradiation (AM 1.5). The material also shows broad-spectrum antimicrobial and antiviral properties against coronaviruses (including the COVID-19 virus), with a reduction rate of 99.99% after 20 min; Gram-positive and Gram-negative bacteria, with a reduction rate of 99.99% after 1 h; fungi, with a reduction rate of 98.4% after 1 h; and multidrug-resistant bacteria, against which it produces a 0.22 cm inhibition ring after 12 h.

## 1. Introduction

Excessive energy consumption and carbon emissions have led to global climate warming [1,2], thus also causing an increase in diseases associated with heat stress and ultimately impeding the sustainable development of human society [3,4]. Passive radiative cooling, which utilizes enhanced reflection in the UV–Vis–NIR wavelength range (0.3–2.5 µm) [5,6,7] and emission in the MIR range (atmospheric window, 8–13 µm) [8,9,10], boasts near-zero energy cost and carbon emissions and has become a promising research direction as part of efforts to replace traditional energy-thirsty air conditioning. Radiative cooling technology can be used in solar cells and fluid-based cooling systems [11,12,13]; it can also be applied in the textile industry for thermal management [5,9,14,15,16,17]. However, most reported radiative cooling materials are made from non-degradable polymers or nanoparticles containing heavy metals [5,16,17], thereby resulting in potential environmental hazards.

We have previously reported the successful development of manufacturing processes for degradable PLA/PHBV textiles [18,19], and were the first to demonstrate their antimicrobial properties—the active ingredient being Poly(3-hydroxybutyrate) (PHB) oligomer [20,21]. In this work, we further investigate one of the homologs, polylactic acid oligomer (PLAO), illustrating that it exhibits a radiative cooling effect when crystallized into a microsphere with a nanotexture, while maintaining effective antimicrobial properties similar to PHB oligomers. The relatively low degree of polymerization (DP) and low molecular weight of PLAO make it much easier to crystallize and form into micro-/nanostructures compared to macromolecular PLA, while the greater number of carboxyl end groups with H^+^ in PLAO per unit mass enables more efficient microbe elimination by altering cellular pH. PLAOs with different DPs and states are synthesized as solid and liquid PLAO coating materials for both radiative cooling and antimicrobial applications.

Compared with previously reported polymeric radiative coatings, the advantages of the PLAO coatings reported in this work are due to the equivalent or higher reflectivity (86.54% in the solar spectrum) and emissivity (92.58% in the mid-infrared range), and their distinct antimicrobial properties (a reduction rate of 99.99% against bacteria and coronaviruses) and biodegradability (99.5% after 28 days). Also, the facile solvent evaporation process for preparing PLAO hexagonal microspheres (PLAOHM) coatings endows this technology with improved industrial viability. In this work, we offer a green one-stop solution for preparing a cooling and antimicrobial coating layer, providing a simple and effective technology for a wide range of healthcare applications.

## 2. Materials and Methods

### 2.1. Synthesis of Polylactic Acid Oligomer (PLAO)

L-Lactic acid agent (L-Lactic acid: H_2_O = 85:15) (Dieckmann, Hong Kong SAR, China) was added to a double-necked flask, one neck of which was used to introduce nitrogen gas with a velocity of 0.5 L/min for protection, and the other was fitted with a spherical condenser for the discharge of H_2_O. The H_2_O generated through condensation was continuously carried away by the nitrogen gas. The reactant, stirred and heated from 20 °C to 200 °C with a heating rate of 3 °C/min, was then maintained for 4 h to obtain a liquid PLAO (yield: 71.4%) and for a further 8 h to obtain a solid PLAO (yield: 68.8%). The pressure in the reactor was maintained at around 1 atm. After the reaction was finished, the temperature was reduced to normal, and all the reaction products were collected.

### 2.2. Antimicrobial Finishing of PP Nonwoven Fabric

Liquid PLAO was mixed with solvent (PLAO:H_2_O = 1:10) to achieve a homogeneous solution, prior to its application on a PP nonwoven fabric using a double-roll padding process. After evaporation of water by drying, the liquid PLAO-finished PP nonwoven fabric was cooled down and collected under ambient conditions for further antimicrobial tests.

### 2.3. Preparation of Hierarchically Porous PLA Oligomer (HPPLAO) Layer

Solid PLAO was heated to 160 to achieve a liquid state, followed by a coating process on horizonal glass or fabric (PP, PET, or cotton fabric) using a doctor blade. The thickness of solid PLAO can be adjusted by controlling the height of the blade. A solvent (DMSO, ethanol, acetone, ethyl acetate, or hexane) was then dropped onto the surface of the solid PLAO. After solvent evaporation and PLAO crystallization for 12–48 h under ambient conditions, the HPPLAO formed as a white coating.

### 2.4. Characterization

A UV–Vis–NIR spectrometer (Perkin Elmer Lambda 1050, Shelton, CT, USA) and FT-IR spectrometer (Spectrum 100, Perkin Elmer) with Integrating Sphere (Mid-IR IntegratIR, PIKE, Hong Kong SAR, China) were used to test the reflectivity and transmissivity of the radiative cooling device/fabrics in the UV–Vis–NIR range (0.25–2.5 μm) and MIR range (4–15 μm), respectively.

**Figure A1:**
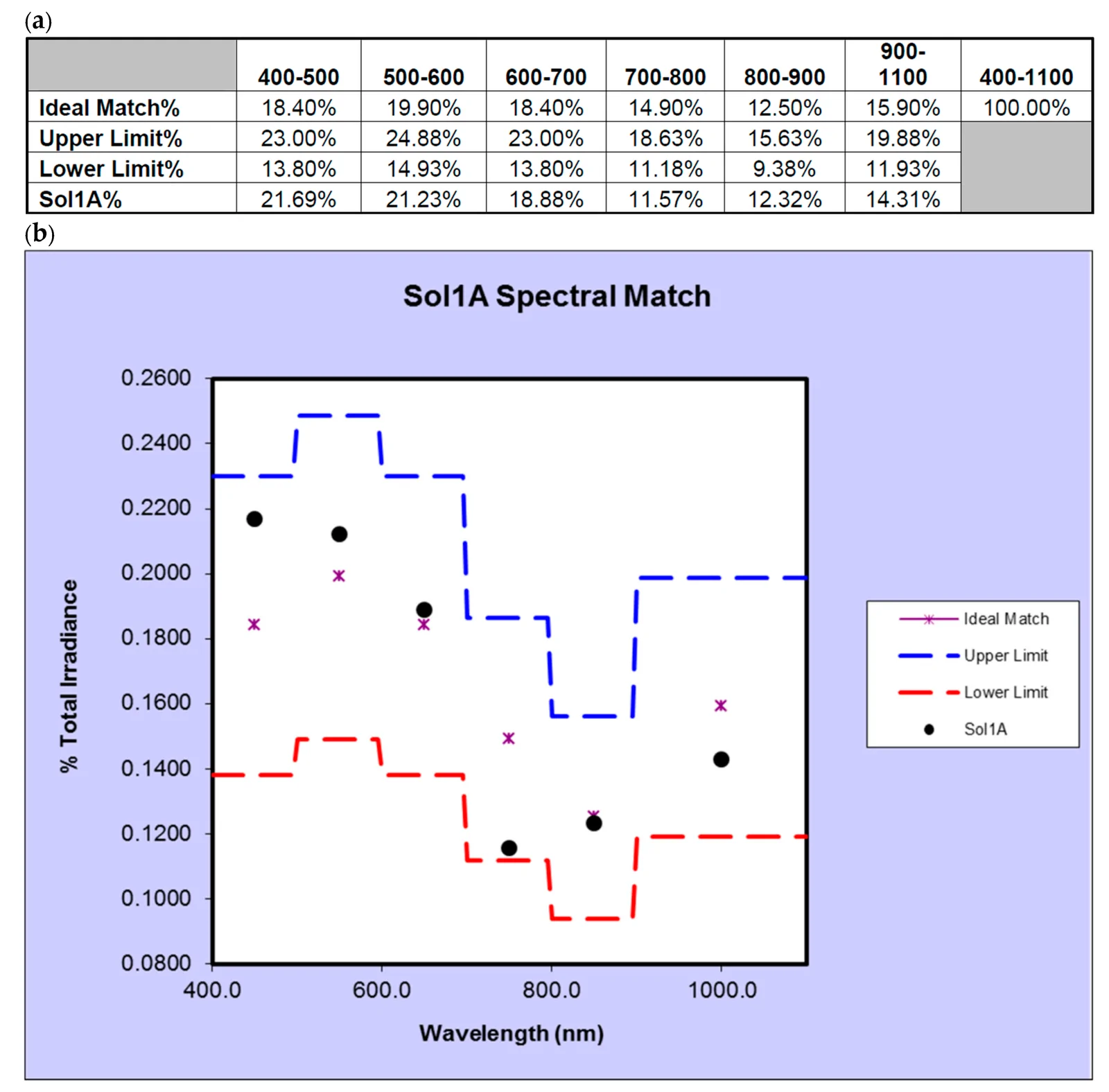
Spectral match report of solar simulator (Oriel^®^ Sol1A™ Class ABB systems, NEWPORT). (**a**) Spectral match data. (**b**) Schematic spectral match of solar simulator.

The radiative cooling tests were carried out under a solar simulator (Oriel^®^ Sol1A™ Class ABB systems, NEWPORT, Irvine, CA, USA) with a power of 1600 W [22] (Figure A1). This system used a xenon lamp with a proprietary filter to simulate the solar spectrum and controlled irradiance through power regulation and reference cell calibration to ensure there was no loss of intensity over time, while achieving a standard 1 SUN (1000 W/m^2^) on the samples and working surface. The sample was placed 27 cm below the radiation source. Ambient temperature and humidity were set to 20 °C and 65% RH.

A thermal imaging camera (E33, FLIR, Hong Kong SAR, China) and thermocouple (Multi-Channel Temperature Meter, AT4516, Anbai, Hong Kong SAR, China) were deployed to measure the temperature of the sample and the surrounding environment. SEM (T3000, Hitachi, Hong Kong SAR, China) was applied to characterize the morphology of HPPLAO. The roughness of the transparent and opaque samples was measured using an Optical Profiling System (New Zygo NexView, Middlefield, CT, USA) and a 3D Optical Measuring System (Alicona IFM G4, Graz, Austria), respectively.

The molecular weight and molecular weight distribution of the samples were determined using a gel permeation chromatography/size exclusion chromatography (GPC/SEC) system (Agilent 1260-2, Hong Kong SAR, China), which was calibrated using narrow-dispersity polystyrene (PS) standards. The number-average molecular weight (Mn), weight-average molecular weight (Mw), and polydispersity index (PDI = Mw/Mn) were calculated using the instrument software.

Contact angle measurements were performed on a fully automatic video contact angle goniometer (JY-82C, Chengde Dingsheng, Chengde, China) using the sessile-drop method. A 1 µL droplet of the test liquid was deposited onto the solid PLAO surface, and the contact angle was determined using the height method and the instrument software. Ultrapure water and diiodomethane were used as probe liquids. All measurements were carried out at 21 °C and 42% relative humidity. The surface free energy (SFE) of solid PLAO and its dispersive and polar components were calculated from the water and diiodomethane contact angles using the Owens–Wendt–Rabel–Kaelble (OWRK) model.

### 2.5. Antimicrobial Test

The antifungal activity of liquid PLAO was assessed according to ASTM E2149-20.

Agar diffusion plate tests of solid PLAO against K. pneumoniae and multidrug-resistant S. aureus were implemented according to ISO 20645-2004. The antiviral and antimicrobial properties of liquid PLAO-finished PP nonwoven fabrics were evaluated according to ISO 18184: 2019 and ASTM E2149-20.

## 3. Results and Discussion

### 3.1. Structure, Morphology, and Optical Spectra of PLAOs

**Figure 1:**
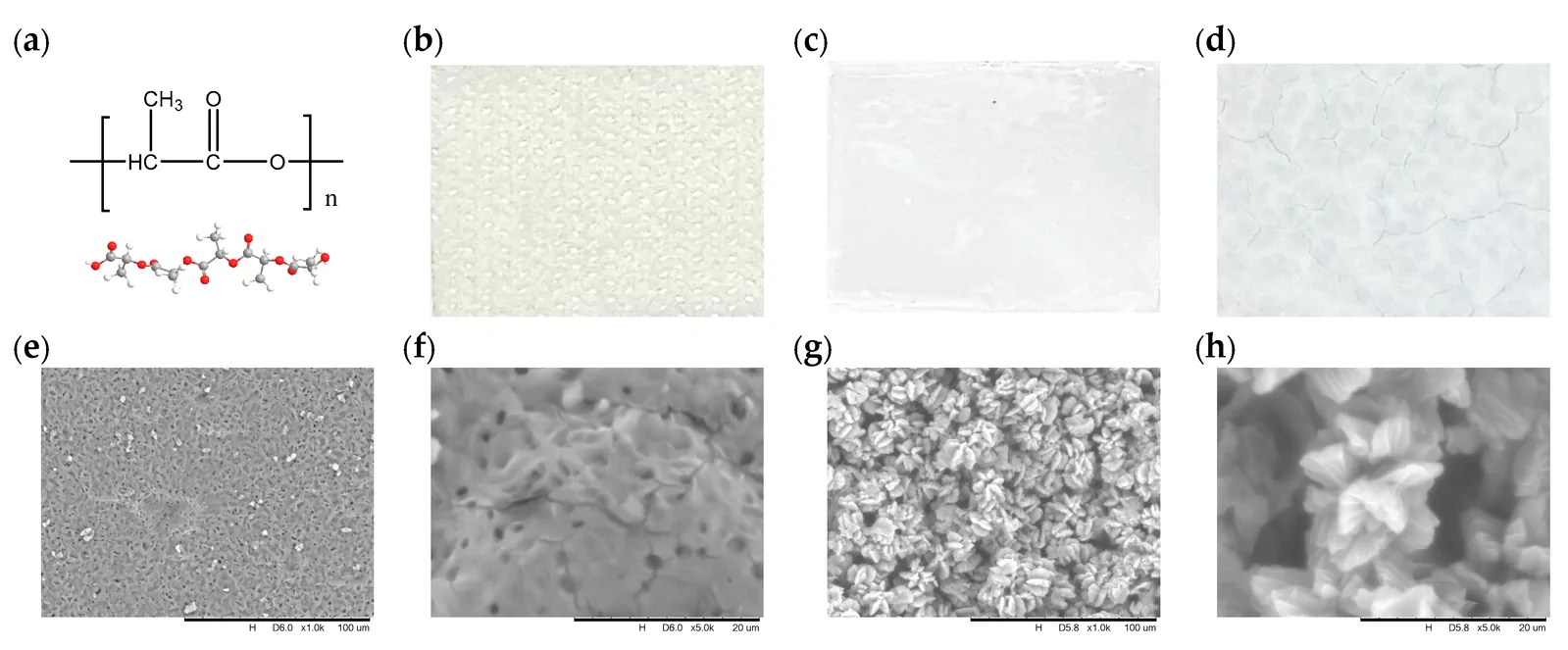

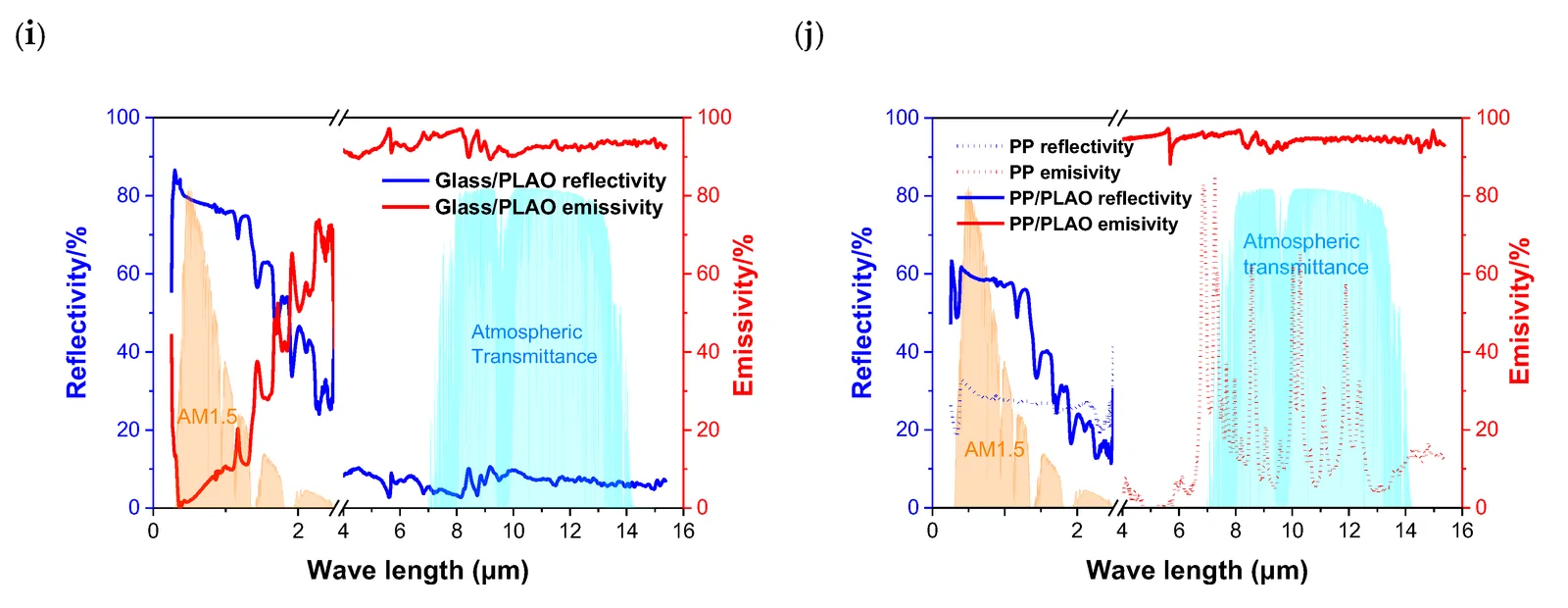
States, morphology variation, and measured optical spectra of PLAOs. (**a**) Liquid PLAO. (**b**) Polypropylene (PP) fabric coated with liquid PLAO. (**c**) Solid PLAO. (**d**) Hierarchically porous PLAO (HPPLAO) formed by ethanol treatment of solid PLAO. (**e**) Surface morphology of HPPLAO (1000X). (**f**) Surface morphology of HPPLAO (5000X). (**g**) PLAO hexagonal microsphere (PLAOHM) in the lower layers of HPPLAO (1000X). (**h**) PLAO hexagonal microspheres in the lower layer of HPPLAO (5000X). (**i**) Reflectivity and emissivity of glass coated with HPPLAO. (**j**) Improvements in reflectivity and emissivity of PP fabric after HPPLAO coating.

Similarly to the synthesis process of the PHB oligomer [20], PLAO was synthesized through simple polymerization of lactic acid monomers. The degree of polymerization (DP) increases with the prolongation of synthesis duration. The state of the PLAO can be adjusted from liquid to solid by varying the polymerizing time. Liquid PLAO is transparent (Figure 1a) and, once diluted with water, can be readily coated onto nonwoven polypropylene (PP) fabrics through a simple soak–pad–dry process. An antimicrobial function can be imparted to the nonwoven PP fabric without any change in appearance (Figure 1b). Solid PLAO is also transparent initially when polymerized for more than 8 h, but may become semitransparent if moisture is absorbed under ambient conditions (Figure 1c). Following solvent treatment for more than 12 h, crystallization occurs on the surface of solid PLAO, thus forming hierarchically porous PLAO (HPPLAO) (Figure 1d), which contains a nano-/microporous layer (pore size: 300 nm–2 μm, Figure 1e,f) in the surface layer and hexagonal microspheres (PLAOHMs) (with microsphere diameters of about 16 μm) with a nanoscale texture (texture size: about 300 nm) (Figure 1g,h) in the sublayer. As can be seen from Figure 1i, the glass–HPPLAO device has a high averaged reflectivity of about 80% in the main solar spectrum of 0.3–1.4 μm (i.e., it reduces the absorption of sunlight), while possessing a relatively high emissivity of about 90% in the mid-infrared (MIR) range of 4–15 μm (i.e., it increases the power radiated out of the device), thus potentially exhibiting a cooling effect. PLAO itself is transparent and rarely absorbs/reflects UV-Vis light, whereas the HPPLAO with micro-/nanostructure can scatter UV-Vis light effectively through multiple scattering effects, thus achieving high reflectivity in the solar spectrum. Meanwhile, the HPPLAO layer is thick enough (0.8 mm) to prevent the transmission of MIR light, resulting in the glass–HPPLAO device exhibiting high emissivity. Similarly, the HPPLAO coating can impart the same property to polypropylene (PP) nonwoven fabric. The short dotted line in Figure 1j shows the low reflectivity and emissivity of the original PP nonwoven fabric, which is used for personal protective equipment (e.g., protective clothing for medical and sanitary use). When an HPPLAO layer is coated onto a PP nonwoven fabric substrate, the reflectivity doubles from 30% to about 60% in the main solar spectrum range, and the emissivity nearly quadruples from 20% to about 80% in the atmospheric window range of 7–14 μm. These remarkable improvements in reflectivity and emissivity indicate that PP–HPPLAO fabric may have a potential cooling effect.

### 3.2. Factors Influencing Morphology and Optical Spectra

#### 3.2.1. Solvent Treatments

**Figure 2:**
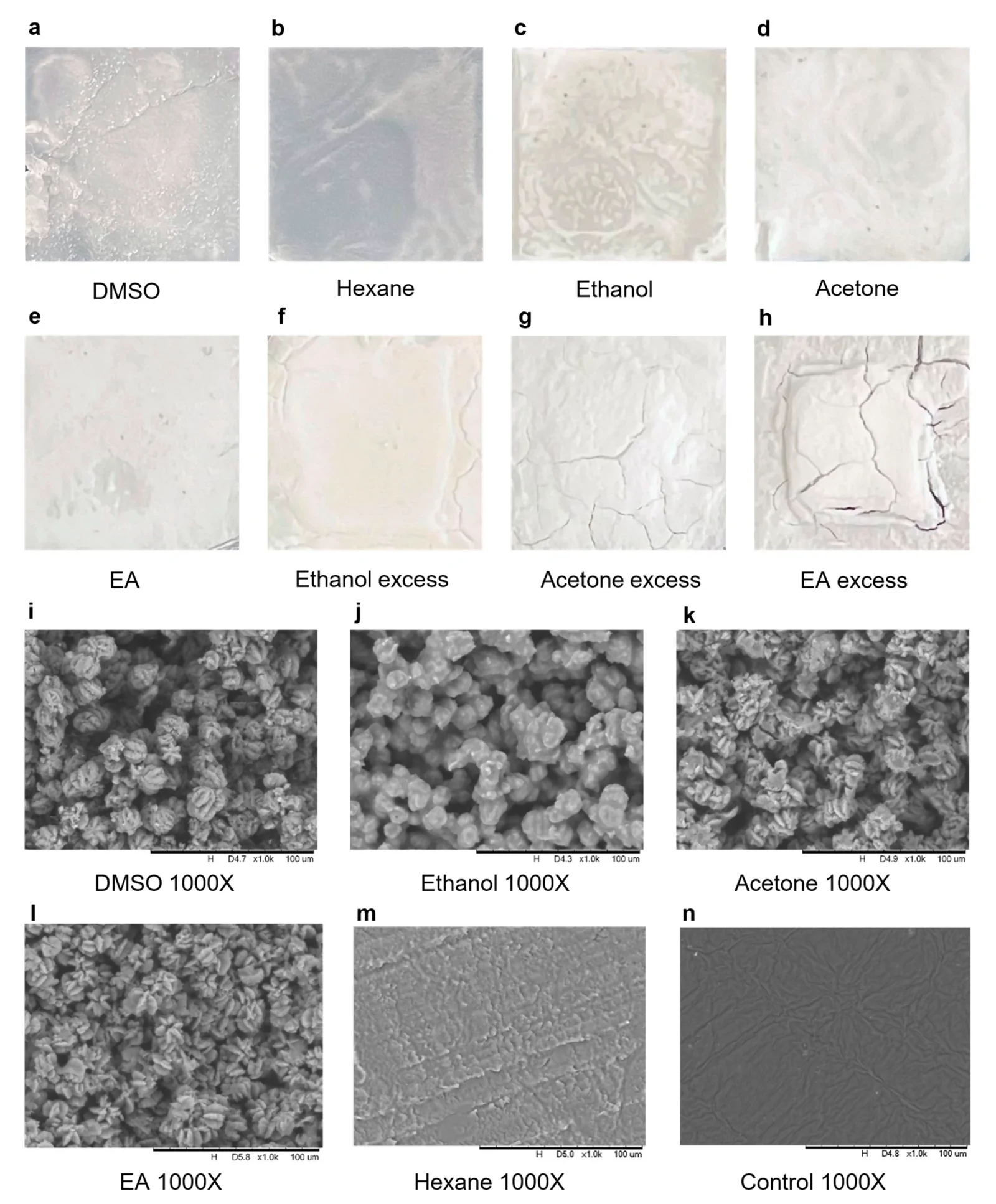
Preparation of HPPLAO through different solvent treatments. (**a**–**e**) HPPLAO prepared using 150 μL of dimethyl sulfoxide (DMSO), hexane, ethanol, acetone, or ethyl acetate (EA), respectively. (**f**–**h**) HPPLAO prepared using excess ethanol, acetone, or EA to fully immerse solid PLAO. (**i**–**l**) PLAO hexagonal microspheres prepared using DMSO, ethanol, acetone, or EA. (**m**) Solid PLAO after hexane treatment. (**n**) Solid PLAO without solvent treatment.

As HPPLAO is prepared through soaking in and evaporation of solvents, the effect of polarity on the formation of HPPLAO was investigated using five solvents ranging from high- to low-polarity, i.e., dimethyl sulfoxide (DMSO), ethanol, acetone, ethyl acetate (EA) and hexane. Figure 2a–e show the appearance of HPPLAO after treatment using different solvents, wherein DMSO- and hexane-treated HPPLAO are more transparent than the samples treated using ethanol, acetone, and EA. The high boiling point of DMSO (189 °C) makes it difficult to vaporize, thus opaque HPPLAO films are formed. Furthermore, the polarity of hexane (no polar group and low polarity) is different from that of PLAO (many polar ester groups and high polarity), leading to incompatibility and preventing the interfacial interactions needed to form HPPLAO. In contrast, ethanol, acetone, and EA have approximately the same polarity as PLAO, so these solvents are compatible with PLAO during the soaking process. Following the ambient evaporation of the solvent and the crystallization of PLAO, an opaque HPPLAO forms with a porous surface layer and a PLAOHM sublayer. Excess quantities of solvents were applied to PLAO in order to investigate the effect of solvent quantity on the formation of HPPLAO. Figure 2f–h show that a larger quantity of solvent can promote the formation of HPPLAO and increase whiteness.

Beyond surface appearance, solvents can also influence the structure of PLAOHM. Figure 2i–n illustrate the different morphologies of PLAOHMs prepared using different solvents, wherein the edges of the hexagonal microspheres are clearer in the PLAO treated using DMSO (though this took over a week to form), acetone, and EA, compared with PLAO treated using ethanol. It can be deduced that the higher moisture absorption of ethanol leads to the partial hydrolysis of HPPLAO, resulting in the loss of the clear hexagonal edges.

#### 3.2.2. Substrates

**Figure 3:**
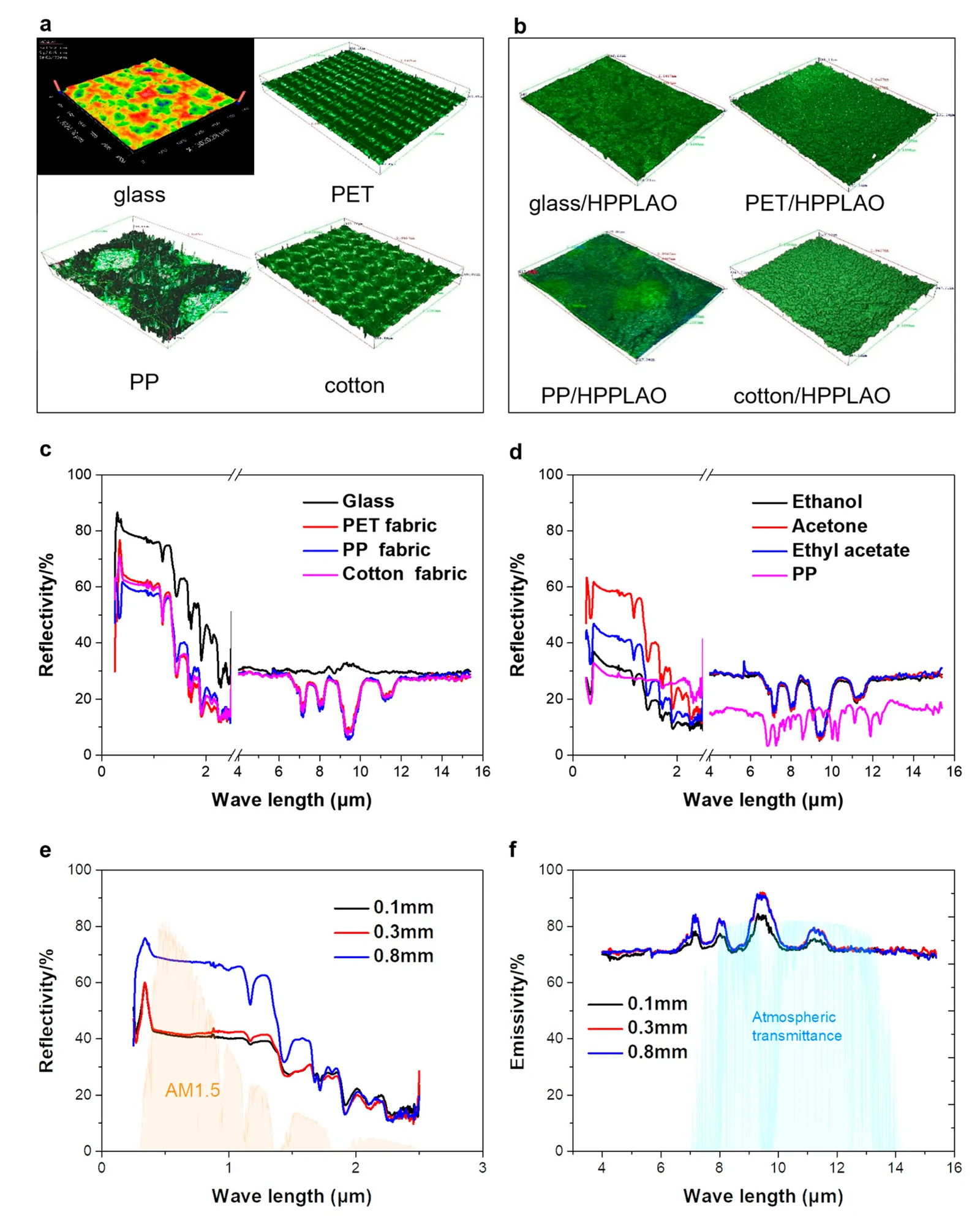
Factors influencing reflectivity and emissivity of HPPLAO. (**a**) Roughnesses of glass (0.066 μm), polyethylene terephthalate (PET) fabric (23.808 μm), polypropylene (PP) nonwoven fabric (183.763 μm), and cotton fabric (49.834 μm). (**b**) Roughnesses of glass–HPPLAO device (13.324 μm), PET–HPPLAO fabric (31.549 μm), PP–HPPLAO fabric (7.396 μm), and cotton–HPPLAO fabric (49.227 μm). (**c**) Effect of substrate on reflectivity of HPPLAO. (**d**) Effects of solvents on reflectivity of PP–HPPLAO fabric. (**e**) Effect of thickness on reflectivity of cotton–HPPLAO fabric. (**f**) Effect of thickness on emissivity of cotton–HPPLAO fabric.

In this work, four kinds of substrates were used to support HPPLAO, which is rigid if untreated, including glass, polyethylene terephthalate (PET) woven fabric, polypropylene (PP) nonwoven fabric, and cotton woven fabric. The roughnesses of different substrates are shown in Figure 3a, while the roughnesses of HPPLAOs based on different substrates are shown in Figure 3b. The order of substrate roughnesses is glass < PET < cotton < PP, whereas the roughness of HPPLAO-based devices/fabrics is glass < PP < PET < cotton, indicating that the roughness of PP decreased remarkably following HPPLAO coating. The reflectivities of HPPLAOs on different substrates are demonstrated in Figure 3c, showing that the reflectivity of HPPLAO is much higher if the roughness of the corresponding substrate is lower. It can be deduced that a substrate with a smooth surface facilitates the formation of more PLAOHMs, which can reflect light more effectively.

As shown in Figure 3d, the PP fabric has the lowest reflectivity in the UV–Vis–NIR range (0.25–1.1 μm) and MIR range (4–15 μm). PP–HPPLAO fabrics prepared by the evaporation of different solvents possess higher reflectivity than the PP fabric, wherein acetone and EA are more effective than ethanol. It can be deduced that the polarity of ethanol is much higher than that of the PP fabric, thus the PP substrate cannot retain ethanol for long enough to form HPPLAO. Conversely, acetone has a similar polarity to the PP fabric and can stay on the substrate long enough to form HPPLAO, which contributes to its higher reflectivity.

#### 3.2.3. Thickness

As shown in Figure 3e,f, the reflectivity of the cotton–HPPLAO fabric increases remarkably from 40% to 70% in the visible spectrum when the thickness rises from 0.1 mm to 0.8 mm. Also, the thickness of HPPLAO slightly improves the emissivity of the cotton–HPPLAO fabric in the MIR range. These results illustrate that a thicker HPPLAO layer can achieve a higher reflectivity in the UV–Vis–NIR range and higher emissivity in the MIR range.

### 3.3. Radiative Cooling

**Figure 4:**
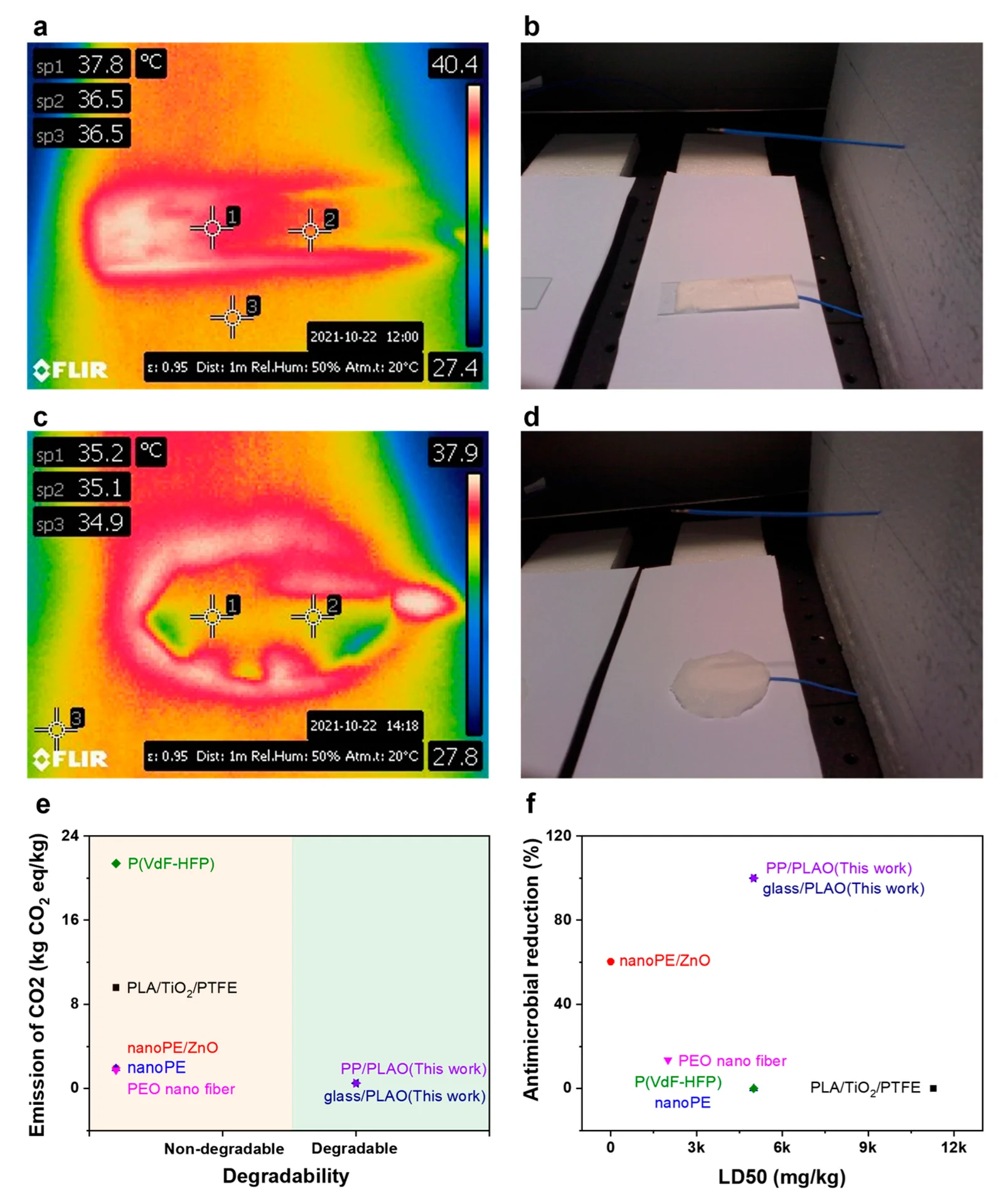
Radiative cooling test for cooling devices/fabrics using thermal images. (**a**) Thermal image of glass–HPPLAO cooling device under AM1.5 solar spectrum irradiation. (**b**) Radiative cooling test setup for glass–HPPLAO device. (**c**) Thermal image of PP–HPPLAO cooling fabric under AM1.5 solar spectrum irradiation. (**d**) Radiative cooling test setup for PP–HPPLAO fabric. (**e**) Comparison of glass–HPPLAO cooling device and PP–HPPLAO cooling fabric with other types of cooling devices/fabrics [5,14,17,23,24] in terms of CO_2_ emissions and degradability. (**f**) Comparison of glass–HPPLAO cooling device and PP–HPPLAO cooling fabric with other types of cooling devices/fabrics [5,14,17,23,24] in terms of antimicrobial performance against *E. coli* and LD50 (rat, oral).

To assess the cooling effect of HPPLAO, the glass–HPPLAO device and the PP–HPPLAO fabric were tested under solar spectrum irradiation (AM1.5) using a thermal imaging camera and thermocouples (Figure 4a–d). The thermal imaging camera was used to record the surface temperature of the sample and of paper, while the thermocouples were used to record the temperature below/above the sample. The detailed temperature of each sample is shown in Table 1. Without an HPPLAO coating, the temperature below the glass was 39.6 °C after solar spectrum irradiation for 3 min, whereas the temperature below the glass–HPPLAO was only 32.8 °C—a temperature reduction of 6.8 °C. Similarly, compared to the PP nonwoven fabric, the PP–HPPLAO fabric achieves a temperature reduction of 4.3 °C at the position below the sample thanks to the cooling effect of HPPLAO.

**Figure A2:**
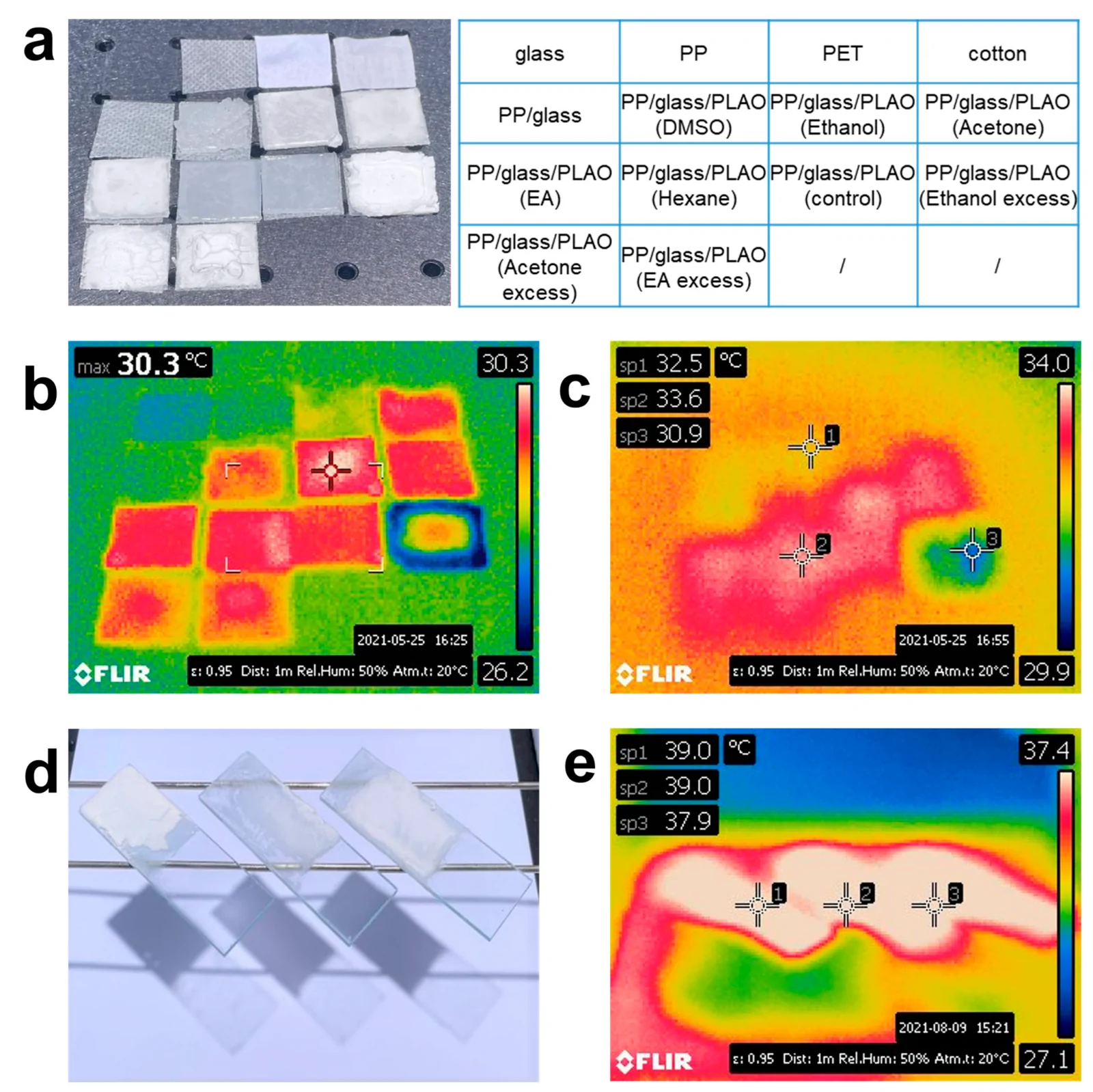
Radiative cooling test using thermal images. (**a**) Cooling materials/devices based on glass, fabrics, and HPPLAO. (**b**) Thermal image of cooling materials/devices under AM1.5 solar irradiation for 10 min. (**c**) Thermal image of cooling materials/devices under AM1.5 solar irradiation for 40 min. (**d**) Setup for assessing cooling effect of glass–HPPLAO cooling devices. (**e**) Thermal image of glass–HPPLAO cooling devices under AM1.5 solar irradiation for 30 min.

**Figure A3:**
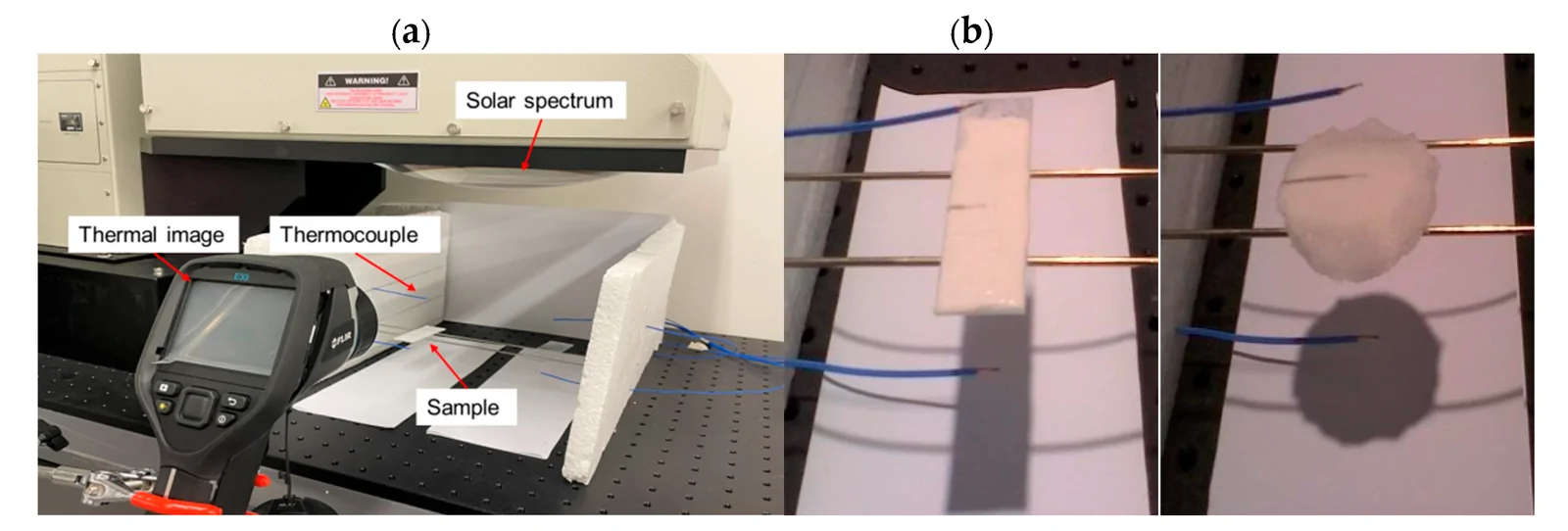

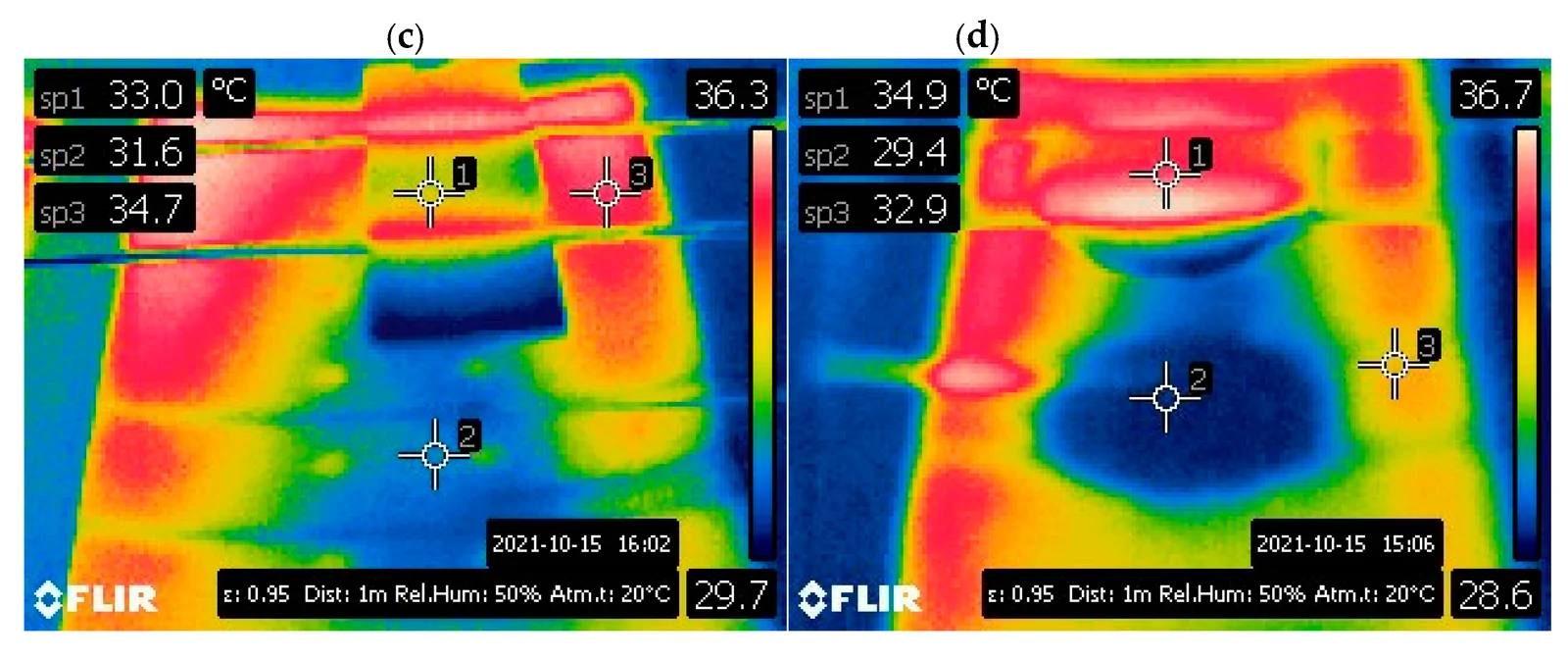
Radiative cooling test using thermal images. (**a**) Overall view of cooling test setup. (**b**) Radiative cooling test setup for glass–HPPLAO device and PP–HPPLAO fabric. (**c**) Thermal image of glass–HPPLAO cooling devices under AM1.5 solar irradiation for 30 min. (**d**) Thermal image of PP–HPPLAO cooling fabric under AM1.5 solar irradiation for 30 min.

Radiative cooling effect can also be observed through the sunlight exposure experiment on impending sample coated with HPPLAO, showing temperature reduction of up to 14.2 °C (Figure A2 and Figure A3, Table A1 and Table A2).

The carbon emissions, biodegradability, antimicrobial properties, and biological safety of the glass–HPPLAO cooling device and PP–HPPLAO cooling fabric are compared with other types of cooling devices/fabrics (Figure 4e,f), including radiative cooling fabrics made by TiO_2_-doped PLA with a PTFE layer [17], a ZnO-doped PE textile [14], a nanoporous polyethylene textile [24], a film based on polyethylene oxide (PEO) nanofibers [23], and a hierarchically porous P(VdF-HFP) film [5].

Figure 4e shows that glass–HPPLAO cooling devices and PP–HPPLAO cooling fabrics are eco-friendly and functional due to the degradability of HPPLAO (99.5% degradation rate after 28 days according to OECD 302B: Inherent Biodegradability: Zahn-Wellens), and their low CO_2_ emissions at 0.5 kg CO_2_ eq/kg compared with other types of cooling devices/textiles [25,26,27,28,29]. Therefore, the radiative cooling properties and eco-friendliness of glass–HPPLAO devices and PP–HPPLAO fabrics are comparable to or better than similar films/fabrics reported previously.

It can be seen from Figure 4f that glass–HPPLAO cooling devices and PP–HPPLAO cooling fabrics exhibit 99.99% antimicrobial reduction against E. Colis, which is significantly higher than that of other types of cooling devices/textiles. Meanwhile, their LD50 values (the dose causing the death of 50% of test animals) are 5000 mg/kg, indicating a higher level of biological safety than devices that contain ZnO and PEO.

Together with their simple preparation processes (relying on a one-step solvent evaporation method) and low cost (due to the raw materials, such as lactic acid and ethanol, being cheap to procure), the cooling devices/fabrics based on HPPLAO are a promising cooling solution in applications with environmental, safety, hygiene, and health requirements such as curtains, tents, umbrellas, etc.

Additionally, HPPLAO prepared by the surficial evaporation of excess ethanol from solid PLAO shows lower temperatures under solar spectrum irradiation (10–40 min) than other materials or fabrics, e.g., glass, PP, PET, cotton, etc. (Figure A2a–c), illustrating the cooling effect generated by the high-reflectivity micro-/nanostructure of HPPLAO. To simulate a sunshade device/fabric, the cooling devices/fabrics were set at a height of 5 cm relative to paper laid on a flat surface. Compared with HPPLAO on the fabric substrate, the glass–HPPLAO device can achieve a temperature drop of 14.2 °C (upper surficial temperature of sample minus temperature of respective shadow on paper, Figure A2d,e and Table A1), exhibiting an excellent radiative cooling effect if used as a sunshade.

In the remote radiative cooling test for the glass–HPPLAO device and PP–HPPLAO fabric, the temperature differences between samples above (2 cm) and below (4 cm) were measured using a thermocouple (Figure A3 and Table A2), and these materials exhibited obvious temperature drops of 3 °C and 2 °C under solar spectrum irradiation for 30 min. These temperature drop values are likely to increase if the sizes of the device/fabric are increased, as a larger area of device/fabric can prevent air convection and energy exchange more efficiently.

### 3.4. Broad-Spectrum Antimicrobial Properties of PLAO

**Figure 5:**
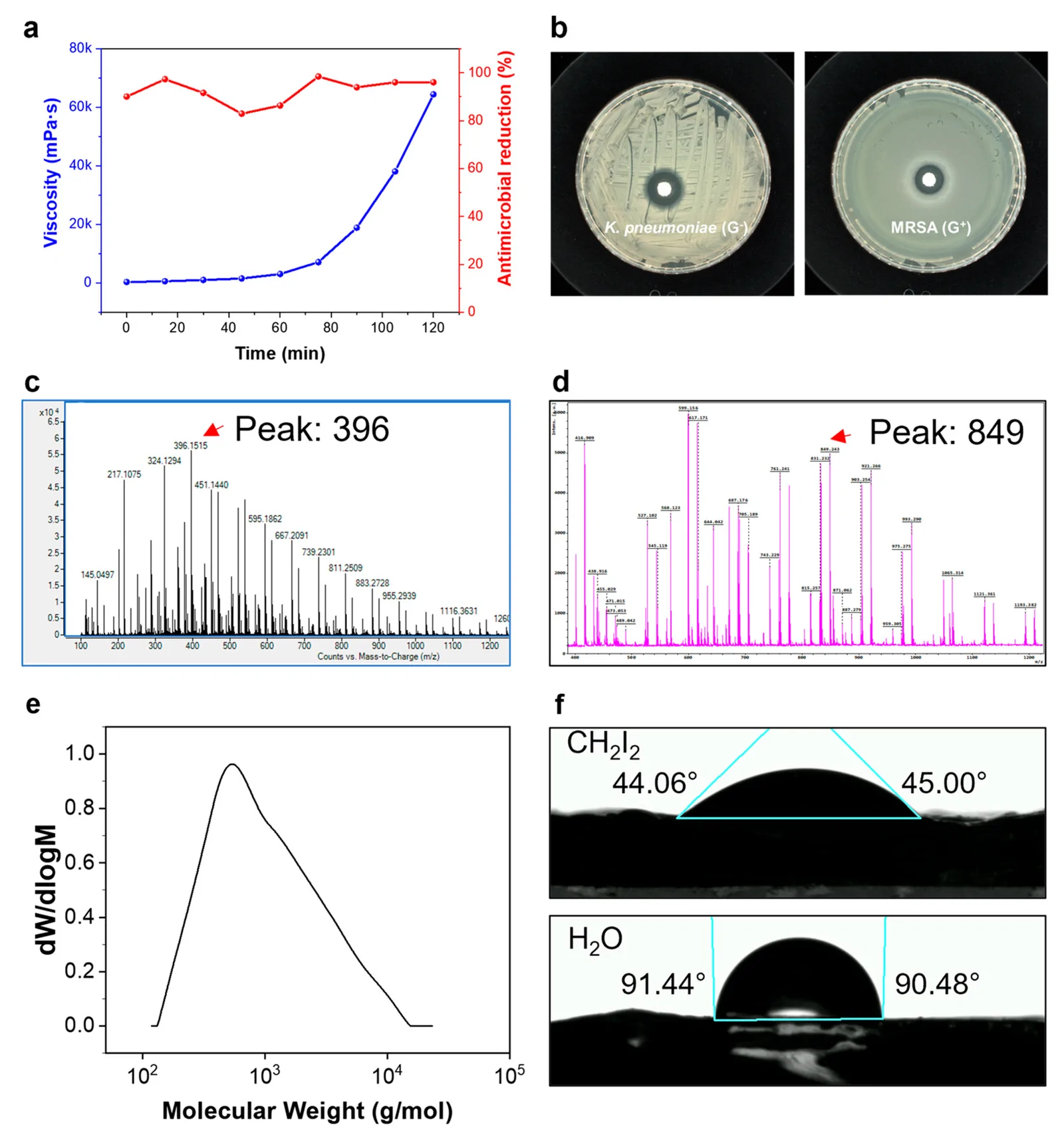
PLAO with antimicrobial function. (**a**) Relationship among antifungal reduction, viscosity, and synthesizing time of liquid PLAO. (**b**) Antibacterial properties of solid PLAO. (**c**) Mass spectrum of liquid PLAO. (**d**) Mass spectrum of solid PLAO. (**e**) GPC/SEC data for solid PLAO. (**f**) Contact angles of CH_2_I_2_ and H_2_O on solid PLAO.

Figure 5a shows the relationship among antifungal reduction against *C. albicans* after 1 h, viscosity, and polymerizing time of liquid PLAO. The viscosity of liquid PLAO increases slightly in the first hour, then rises sharply in the second hour, illustrating the generation of an oligomer with a higher DP. The number-average molecular weight M_n_ of liquid PLAO is 432 at 120 min with its highest peak at 396 (Figure 5c). The antifungal reduction ranges from 82.9% to 98.4% and does not change obviously with the increase in viscosity; the main component and DP of PLAO did not change much with a polymerizing time of 2 h. Table 2 summarizes the broad-spectrum antimicrobial properties of liquid PLAO and nonwoven PP fabrics coated with it against coronaviruses, Gram-positive bacteria, Gram-negative bacteria, and fungi. The antiviral reductions against SARS-CoV-2 (COVID-19 virus) of 1% and 2% PLAO aqueous solutions can reach 99.99% in 20 min and 1.5 h, respectively. The antiviral effects of PLAO-coated nonwoven PP fabric against H3N2 virus increase with longer contact time to reach a reduction maximum of 99.99% following a contact time of 2 h. The liquid PLAO-coated nonwoven PP fabrics also show strong antimicrobial properties against other microbes, especially Gram-positive and Gram-negative bacteria (99.99%). Considering the fact that PLAO is a homolog of a PHB oligomer, its antimicrobial mechanism is probably the same as that of the PHB oligomer, i.e., the destruction of microbes’ biofilm, cell membranes, and proteins [20].

Figure 5b exhibits the antibacterial effect of solid PLAO, in which M_n_ equals 644 with a polydispersity index of 2.54 (Figure 5d,e), against *K. pneumoniae* and multidrug-resistant *S. aureus* according to ISO 20645-2004, where inhibition ring widths are 0.27 cm and 0.22 cm (loading density: 0.6 mg/mm^2^), respectively, indicating effective antibacterial performance against Gram-positive, Gram-negative, and multidrug-resistant bacteria. What is more, the total surface free energy of solid PLAO was calculated to be 38.30 mN/m based on the OWRK model and contact angle test (Figure 5f), with a dispersive component of 37.26 mN/m and a polar component of 1.04 mN/m. The surface free energy was therefore dominated by dispersive interactions, while the very low polar component (1.04 mN/m) indicates a low surface polarity, consistent with the relatively high water contact angle, thus reducing bacterial adhesion.

## 4. Conclusions

In conclusion, we demonstrate a coating material based on HPPLAO that simultaneously possesses biodegradability, a radiative cooling effect, and broad-spectrum antimicrobial properties. The radiative cooling effect arises from the hexagonal microsphere geometry and nanotexture of PLAOHM crystals, which can enhance reflectivity in the solar spectrum through a multiple scattering effect and exhibit high emissivity in the mid-infrared atmospheric window due to the vibration modes in PLAO. The optimal number-average molecular weight Mn of PLAOHM crystals to achieve the best radiative cooling effect is 644.

Additionally, owing to its destruction of cell membranes and microbial proteins, the material shows broad-spectrum antimicrobial properties and significant antimicrobial reductions against coronaviruses (including the COVID-19 virus), Gram-positive bacteria, Gram-negative bacteria, multidrug-resistant bacteria, and fungi. The optimal number-average molecular weight Mn of PLAO to achieve best antimicrobial effect is 432.

This work opens a door leading to the use of oligomer crystals in the development of multifunctional, energy-saving, and hygienic materials, encompassing advantages such as lower carbon emissions (low to 1/40), higher biosafety (up to 200 times), and improved eco-friendliness (faster biodegradability) compared with traditional materials and devices described in the literature [5,14,17,23,24]. Though some disadvantages persist, such as thermal and humidity stability, similar multifunctional materials may be discovered by adopting this approach of changing the functional group, degree of polymerization, polarity, crystal size, etc., thus satisfying diverse requirements across a wide range of applications.

The effects of crystallization temperature, humidity, and degree of polymerization on pore size and microsphere diameter will be investigated in future work, thereby furthering understanding of the nucleation and crystallization mechanisms and ultimately establishing relationships between structural dimensions and reflectivity.

## 5. Patents

Publication No.: CN122037738A; Invention Name: Bio-based micro-/nanomaterial refrigeration device with radiation cooling and antibacterial functions, and applications thereof.

## Figures and Tables

**Table 1:** Surface temperature (°C) of devices/fabrics coated with HPPLAO.

Test Position	Glass	Glass–HPPLAO	PP	PP–HPPLAO
Sample above (9 cm)	29.1	27.5	27.3	27.7
Sample	35	32.1	32.2	31.7
Sample below	39.6	32.8	39.5	35.2
Paper under solar spectrum	33.1	33.1	32.8	33.4
Reduced temperature	/	6.8	/	4.3

**Table 2:** Antimicrobial properties of liquid PLAO and liquid PLAO-coated nonwoven PP fabrics.

Sample	Testing Standard and Method	Tested Microbe	Contact Time	Antimicrobial Reduction (%)
1% PLAO	ISO 18184: 2019 and Technical Specification for Disinfection, 2002, China	SARS -CoV-2	20 min	>99.99
2% PLAO			20 min	>99.99
1% PLAO			1.5 h	>99.99
2% PLAO			1.5 h	>99.99
PLAO-coated fabric	ISO 18184: 2019	H3N2	2 h	>99.99
PLAO-coated fabric	ISO 18184: 2019	H3N2	30 min	>81.16
PLAO-coated fabric	ISO 18184: 2019	H3N2	10 min	>54.38
PLAO-coated fabric	ISO 18184: 2019	H3N2	5 min	>42.72
PLAO-coated fabric	ISO 18184: 2019	H1N1	2 h	>95.11
PLAO-coated fabric	ASTM E2149-20	*S. Aureus*	1 h	>99.99
PLAO-coated fabric	ASTM E2149-20	*K. pneumoniae*	1 h	>99.99
PLAO-coated fabric	ASTM E2149-20	*C. albicans*	24 h	81.7%

## Data Availability

The original contributions presented in this study are included in the article. Further inquiries can be directed to the corresponding authors.

## Abbreviations

The following abbreviations are used in this manuscript:

PLAO	polylactic acid oligomer
PHB	poly(3-hydroxybutyrate)
DP	degrees of polymerization
HPPLAO	hierarchically porous polylactic acid oligomer
MIR	mid-infrared
PP	polypropylene
PLAOHM	PLAO hexagonal microsphere
DMSO	dimethyl sulfoxide
EA	ethyl acetate
PET	polyethylene terephthalate
PEO	polyethylene oxide

## Appendix A

**Table A1:** Cooling effect of devices/fabrics based on HPPLAO.

Sample	Location	Ethanol	Acetone	EA	Without PLAO
Glass–HPPLAO 0.8 mm	HPPLAO	44.1	45.3	46	41.6
	Shadow	31.5	31.1	32.1	34.3
	Difference	12.6	14.2	13.9	7.3
PP–HPPLAO 0.8 mm	HPPLAO	44.3	39.8	44.6	38.7
	Shadow	36.8	35.2	36.2	35.2
	Difference	7.5	4.6	8.4	3.5
PET–HPPLAO 0.8 mm	HPPLAO	45.8	40.9	44.4	42.3
	Shadow	37.8	36.5	37.8	36.2
	Difference	8	4.4	6.6	6.1
Cotton–HPPLAO 0.8 mm	HPPLAO	43.7	44.8	45.4	44.3
	Shadow	38.8	37.2	38.8	36.9
	Difference	4.9	7.6	6.6	7.4
Cotton–HPPLAO 0.3 mm	HPPLAO	43.6	44.3	44.4	43.5
	Shadow	38.9	38.1	38.6	37.9
	Difference	4.7	6.2	5.8	5.6
Cotton–HPPLAO 0.1 mm	HPPLAO	43.6	44.1	44.4	43.9
	Shadow	39.2	39	39	38.7
	Difference	4.4	5.1	5.4	5.2

Note: Data obtained after 10 min of solar spectrum irradiation.

**Table A2:** Surface temperature (°C) of impending devices/fabrics coated with HPPLAO.

Test position	Glass	Glass–HPPLAO	PP	PP–HPPLAO
Sample above (2 cm)	32.9	31.2	29.8	28.7
Sample	33.2	37.2	29	33.6
Sample below (4 cm)	31.8	28.2	29.1	26.7
Paper under sample (5 cm)	31.9	32	28.2	28.9
Paper under solar irradiation	34.1	34.5	31.1	32.4
Temp. difference between sample above and below	1.1	3	0.7	2
Temp. difference between paper with and without sample cover	2.2	2.5	2.9	3.5
Temp. difference between sample and paper under sample	1.3	5.2	0.8	4.7

Note: Data obtained after 30 min of solar irradiation.
